# Supplementary figures and images for: The Kv2.2 channel mediates the inhibition of prostaglandin E2 on glucose-stimulated insulin secretion in pancreatic β-cells
Source: eLife. 2025 Mar 3;13:RP97234. doi: 10.7554/eLife.97234 (PMC11875535; doi:10.7554/eLife.97234)

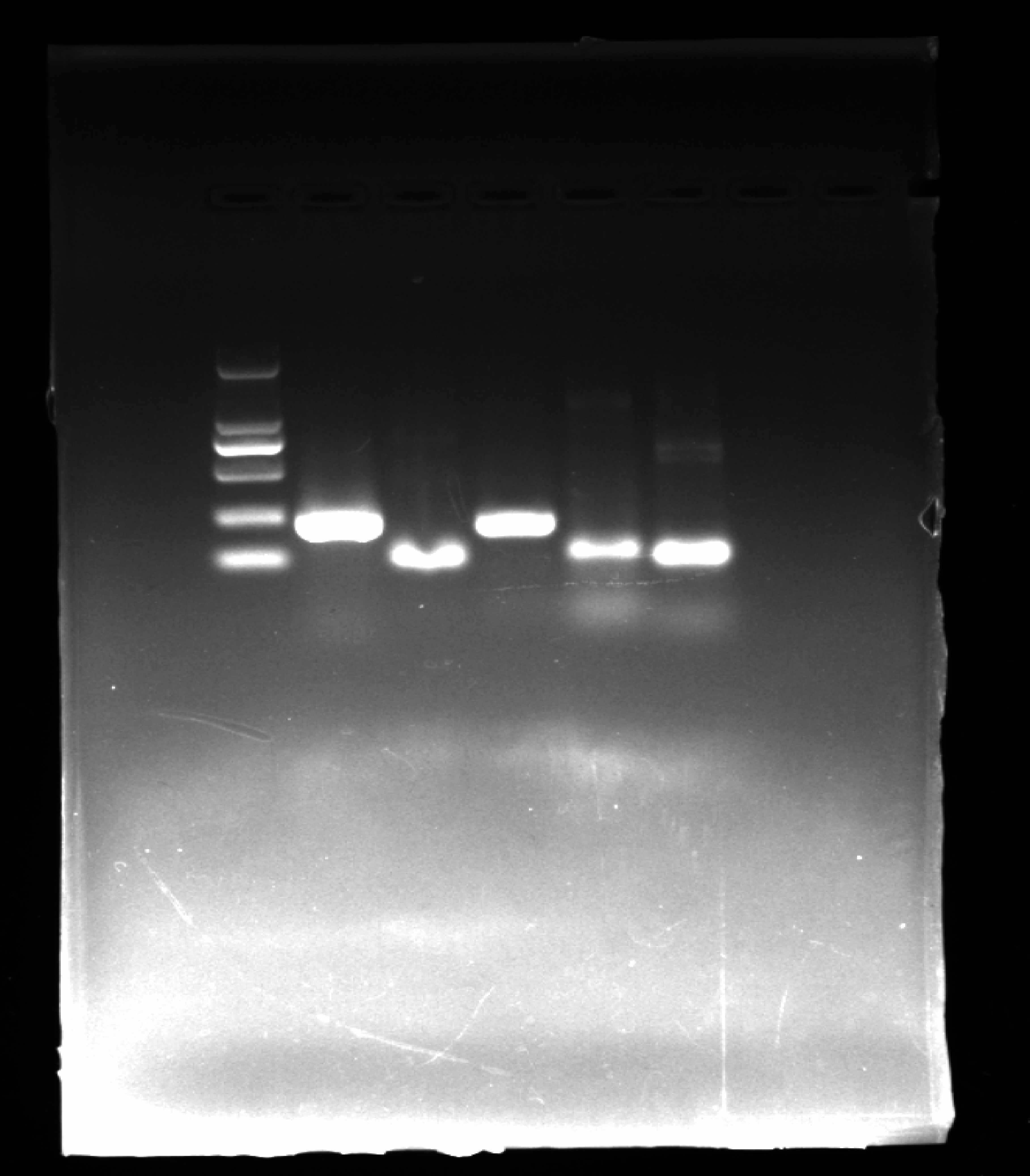

Supplement: Figure 2—source data 2. [file elife-97234-fig2-data2.zip › Full DAN gel image/DNA gel image.tif]

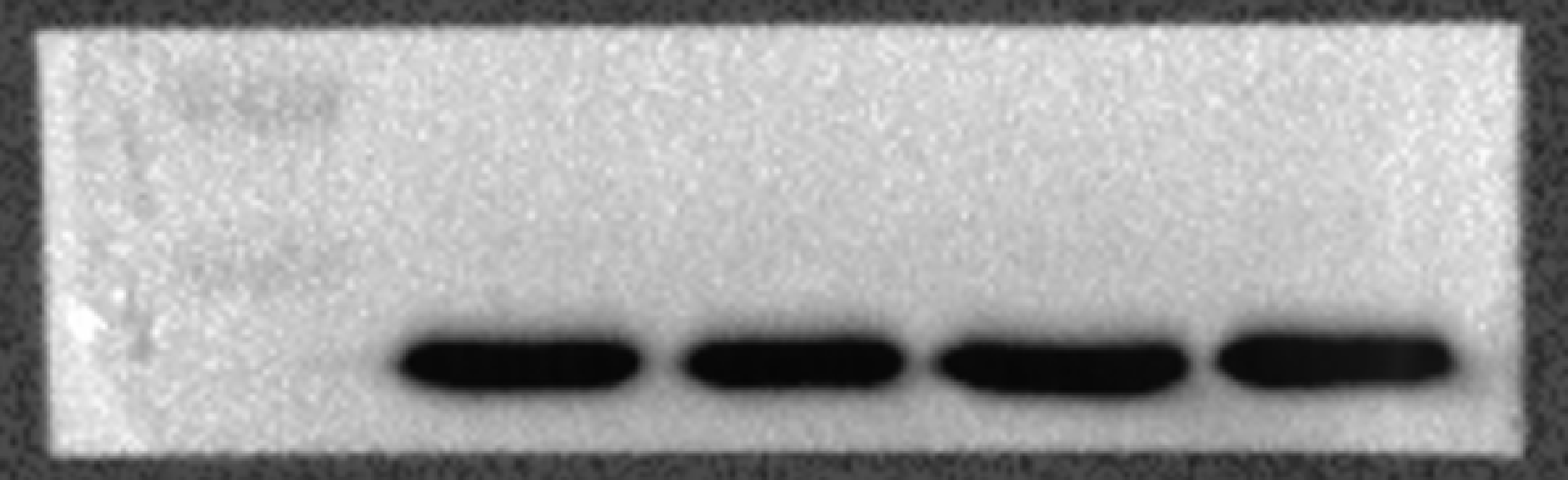

Supplement: Figure 3—source data 2. [file elife-97234-fig3-data2.zip › full blot/GAPDH.tif]

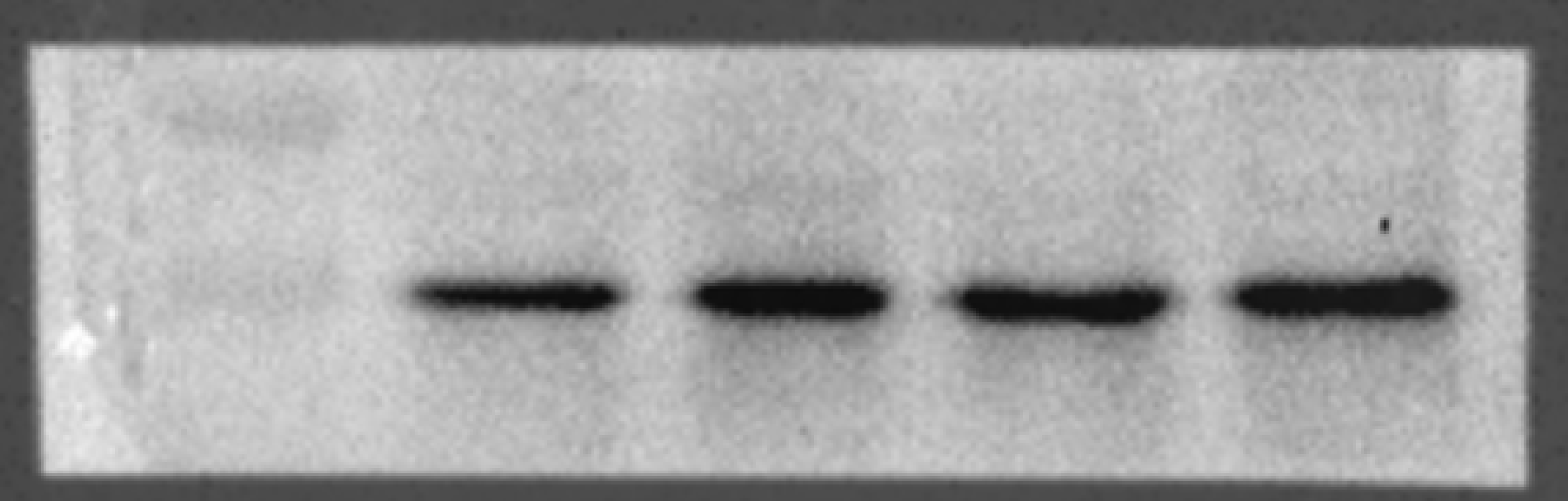

Supplement: Figure 3—source data 2. [file elife-97234-fig3-data2.zip › full blot/p-PKA.tif]

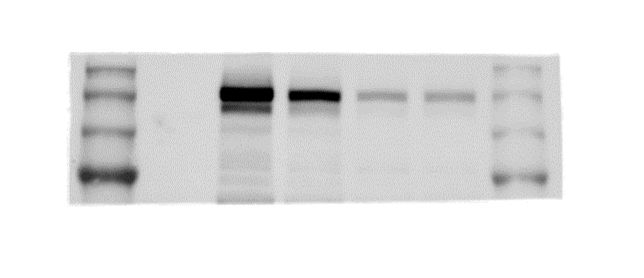

Supplement: Figure 4—source data 2. [file elife-97234-fig4-data2.zip › full blot/Kv2.2.tif]

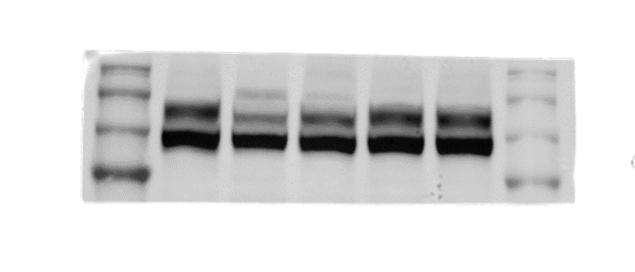

Supplement: Figure 4—source data 2. [file elife-97234-fig4-data2.zip › full blot/Na+-K+ATPase.tif]

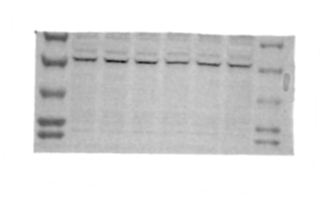

Supplement: Figure 5—figure supplement 1—source data 2. [file elife-97234-fig5-figsupp1-data2.zip › Figure 5-figure supplement 1-sourece data 2/Figure 5-figure supplement 1, Source Data-2-A/EP1 receptor.tif]

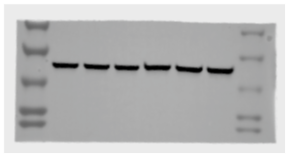

Supplement: Figure 5—figure supplement 1—source data 2. [file elife-97234-fig5-figsupp1-data2.zip › Figure 5-figure supplement 1-sourece data 2/Figure 5-figure supplement 1, Source Data-2-A/GAPDH.tif]

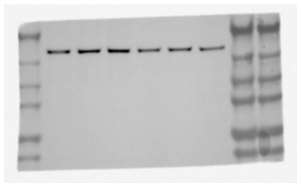

Supplement: Figure 5—figure supplement 1—source data 2. [file elife-97234-fig5-figsupp1-data2.zip › Figure 5-figure supplement 1-sourece data 2/Figure 5-figure supplement 1, Source Data-2-B/EP2 receptor.tif]

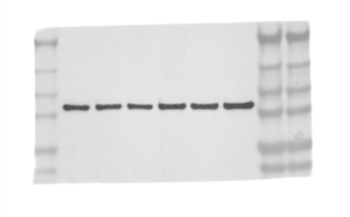

Supplement: Figure 5—figure supplement 1—source data 2. [file elife-97234-fig5-figsupp1-data2.zip › Figure 5-figure supplement 1-sourece data 2/Figure 5-figure supplement 1, Source Data-2-B/GAPDH.tif]

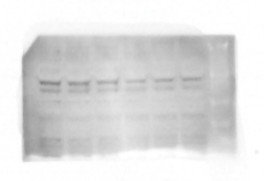

Supplement: Figure 5—figure supplement 1—source data 2. [file elife-97234-fig5-figsupp1-data2.zip › Figure 5-figure supplement 1-sourece data 2/Figure 5-figure supplement 1, Source Data-2-C/EP3 receptor.tif]

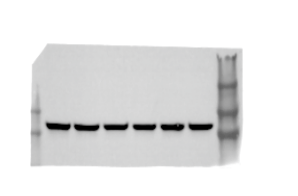

Supplement: Figure 5—figure supplement 1—source data 2. [file elife-97234-fig5-figsupp1-data2.zip › Figure 5-figure supplement 1-sourece data 2/Figure 5-figure supplement 1, Source Data-2-C/GAPDH.tif]

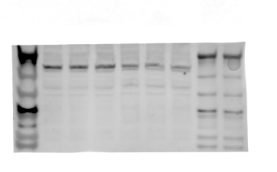

Supplement: Figure 5—figure supplement 1—source data 2. [file elife-97234-fig5-figsupp1-data2.zip › Figure 5-figure supplement 1-sourece data 2/Figure 5-figure supplement 1, Source Data-2-D/EP4 receptor.tif]

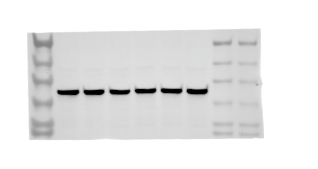

Supplement: Figure 5—figure supplement 1—source data 2. [file elife-97234-fig5-figsupp1-data2.zip › Figure 5-figure supplement 1-sourece data 2/Figure 5-figure supplement 1, Source Data-2-D/GAPDH.tif]

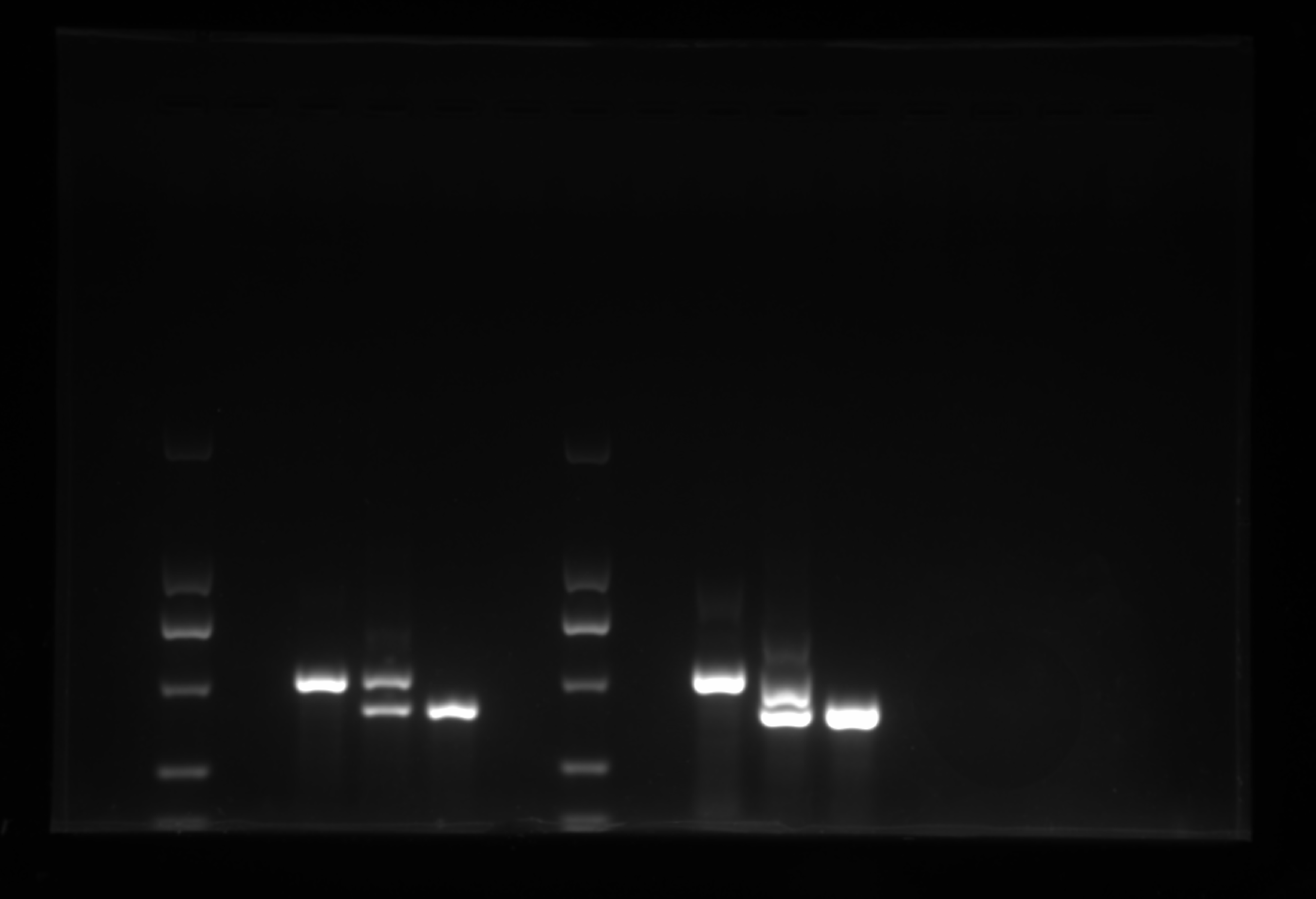

Supplement: Figure 7—figure supplement 1—source data 2. [file elife-97234-fig7-figsupp1-data2.zip › DNA gel image.tif]
