# Supplementary material for: The Kv2.2 channel mediates the inhibition of prostaglandin E2 on glucose-stimulated insulin secretion in pancreatic β-cells
Source: eLife. 2025 Mar 3;13:RP97234. doi: 10.7554/eLife.97234 (PMC11875535; doi:10.7554/eLife.97234)
Supplement: Figure 4—source data 3. [file elife-97234-fig4-data3.zip › full blot with lable.pdf]

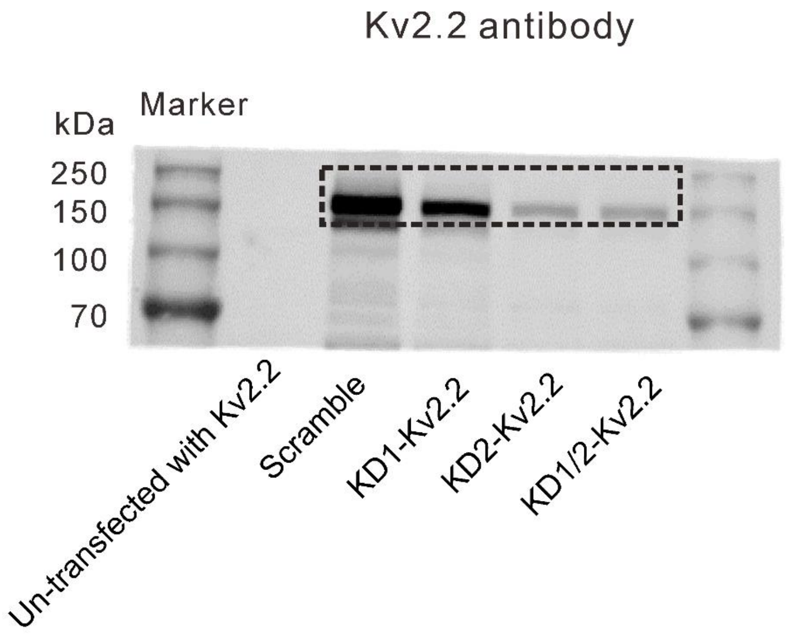

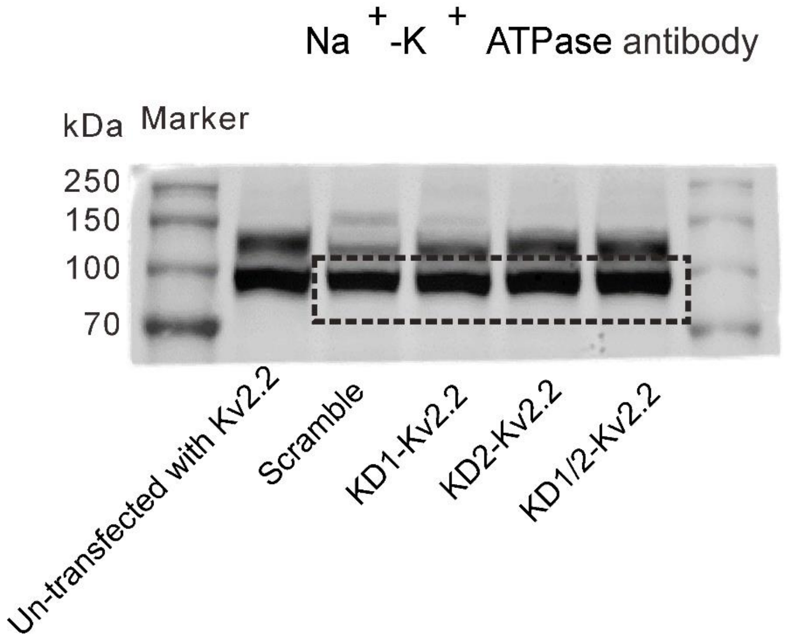

Figure 4C, Source Data 1. Original western blot images corresponding to Figure 4C. The areas enclosed by the dashed lines represent the content shown in Figure 4C.
