# Supplementary material for: The Kv2.2 channel mediates the inhibition of prostaglandin E2 on glucose-stimulated insulin secretion in pancreatic β-cells
Source: eLife. 2025 Mar 3;13:RP97234. doi: 10.7554/eLife.97234 (PMC11875535; doi:10.7554/eLife.97234)
Supplement: Figure 5—figure supplement 1—source data 3. [file elife-97234-fig5-figsupp1-data3.zip › Figure 5-figure supplement 1- Source Data 3.pdf]

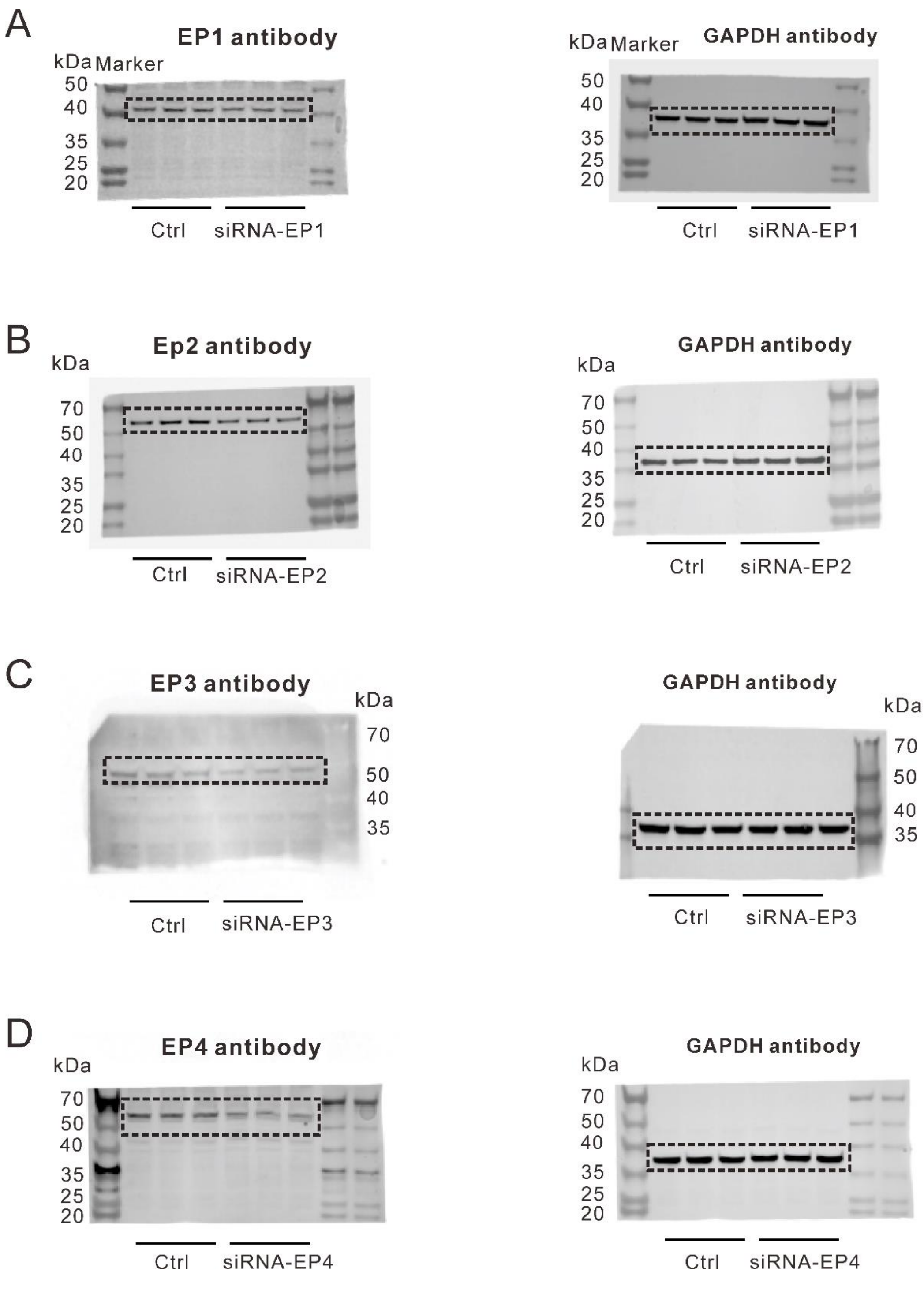

Figure 5-figure supplement 1-Source Data 2. Original western blot images corresponding to Figure 5-figure supplement 1. The areas enclosed by the dashed lines represent the content shown in Figure 5-figure supplement 1 (A~D).
