# Supplementary material for: The Kv2.2 channel mediates the inhibition of prostaglandin E2 on glucose-stimulated insulin secretion in pancreatic β-cells
Source: eLife. 2025 Mar 3;13:RP97234. doi: 10.7554/eLife.97234 (PMC11875535; doi:10.7554/eLife.97234)
Supplement: Figure 7—figure supplement 1—source data 3. [file elife-97234-fig7-figsupp1-data3.zip › Figure 7-figue supplement 1-Source Data 3.pdf]

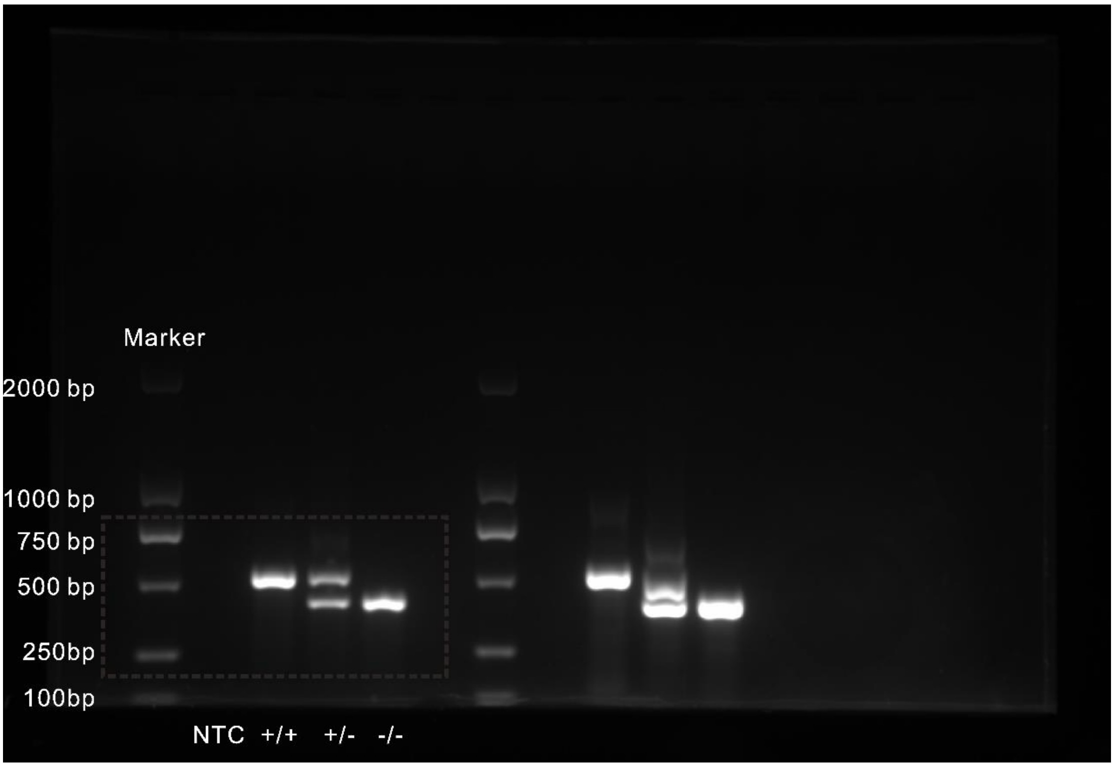

Figure 7-figure supplement 1-Source Data 2. Uncropped DNA gel image for Fig.7-figure supplement 1. The area enclosed by the dashed line represents the content shown in Figure 7-figure supplement 1 A.
